# Flower angle favors pollen export efficiency in the snowdrop *Galanthus nivalis* (Linnaeus, 1753) but not in the lesser celandine *Ficaria verna* (Huds, 1762)

**DOI:** 10.1080/15592324.2022.2163065

**Published:** 2023-01-12

**Authors:** Pavol Prokop, Zuzana Ježová, Michaela Mešková, Viktória Vanerková, Martina Zvaríková, Peter Fedor

**Affiliations:** a Department of Environmental Ecology and Landscape Management, Faculty of Natural Sciences, Comenius University, Bratislava, Slovakia; b Institute of Zoology, Slovak Academy of Sciences, Bratislava, Slovakia

**Keywords:** Bumblebee, pendant flowers, pollinators, radial symmetry

## Abstract

Flower angle is crucially important for accurate pollination and flower protection against abiotic factors. Evolutionary factors shaping floral traits are particularly strong for bilaterally symmetric flowers because these flowers require more pollination accuracy than radially symmetrical flowers. We experimentally investigated the flower angle in the snowdrop’s (*Galanthus nivalis*) radially symmetrical, early-blooming downward flowers. Bumblebees were able to gather significantly more pollen grains from downward flowers than from upward flowers, but female traits (fertility in the field) seem unaffected by flower angle. Similar experiments with radially symmetrical, later flowering Lesser celandine (*Ficaria verna*) upward flowers showed no differences in bees’ abilities to gather pollen in upward vs downward-facing flowers. The downward angle of snowdrop flowers is an adaptation that increases the ability of insects to collect more pollen grains under unfavorable early spring weather conditions when pollinators are scarce.

## Introduction

Major pressures of floral traits in entomophilous plants are pollinators, which facilitate pollen transfer between plants, and plants therefore need to attract them to increase their fitness^1^. Floral traits, however, are further shaped by other biotic factors such as predators, and by abiotic factors, such as temperature or photoperiod.^2^ The resulting interactions between these traits^3,4^ influence an enormous variation of floral traits such as shape, color, size, and orientation,^5,6^ which ultimately enhances the reproductive success of individual plants.^7–10^

Research on flower angles (upward, horizontal, downward) integrates evolutionary approaches to studying both abiotic and biotic influences on floral traits. Regarding the former, horizontal or downward angle serves as a rain protection device, ultimately reducing pollen damage and nectar dilution.^9,11–14^ Considering biotic factors, floral angle influences pollinator attraction,^9,10,15–18^ which consequently enhances plant reproductive success through effective pollen transfer.^9,10,17–20^ The majority of these works investigated the role of flower angle in bilaterally symmetric (zygomorphic) flowers, while research on radially symmetrical (actinomorphic) flowers is much less developed.^10^ Researchers suggest that flower angle in actinomorphic flowers is under weaker selective constraints and pollinator positioning is less crucial than in zygomorphic flowers,^21,22^ because zygomorphic flowers influence pollinator position more precisely to effectively promote physical contact between flowers’ reproductive organs and pollinators’ bodies, which alters pollen removal and deposition.^17,23,24^ We hypothesize that selective constraints favoring the ability of bees to gather pollen mediated through insects in actinomorphic flowers with downward flower angles are similarly important as in zygomorphic flowers because flower angle influences pollinator behavior and availability.^10,25^ Specifically, we predict that bees can gather more pollen grains from downward-facing flowers transfer than from experimentally altered upward-facing flowers. In contrast, naturally upward flowers utilizing different mechanisms of pollen transfer, such as adhesion of pollen grains, do not enhance gathering pollen by experimentally altering downward flower angle.

In this study, we investigated the ability of bees to gather pollen in radially symmetrical downward flowers of snowdrops (*Galanthus nivalis*) and in upward radially symmetrical flowers of the lesser celandine (*Ficaria verna*). Natural, downward snowdrop flowers were found to be less attractive for naive bumblebees (*Bombus terrestris*) than experimentally altered upward flowers.^26^ This suggests that the downward flower angle in the snowdrop primarily did not evolve to attract pollinators, but its original function is different. Lesser celandine was used as a model species with upward radially symmetrical flowers because it occupies similar habitats and blooms early in the spring, similarly to the snowdrop, but its flower angle is upward. We experimentally investigated whether a downward angle may enhance the ability of bees to gather pollen compared with an upward angle using both naturally downward (snowdrop) and upward (lesser celandine) species.

## Methods

### Study species

Snowdrop (Amaryllidaceae) is a perennial, early flowering plant (February–March) occurring in Central Europe, Asia Minor, and the Caucasus.^27^ It reproduces both vegetatively and sexually. Sexual reproduction is mainly accomplished by xenogamy and less by autogamy, which is costly in terms of prolonged flowering and reduced reproductive success.^28^ Snowdrops are pollinated mainly by honey bees and bumblebees,^27^ which are scarce due to low spring temperatures (P. Prokop, unpublished data). Snowdrop has a consistent downward flower angle.

Lesser celandine (Ranunculaceae) is a perennial, early flowering plant (March–April) native to Europe, temperate Asia, and northern Africa.^29^ It reproduces vegetatively via tubers and sexually via seeds. Flowers are yellow, about 2–6 cm wide.^30^ Pollinators are mainly hymenopterans, dipterans, and beetles.^31^ Pollinator availability strongly depends on weather conditions (P. Prokop, pers. obs.). It produces fertile seeds through both cross-pollination and self-pollination,^32^ but the possible reproductive costs of self-pollination are unknown. Lesser celandine has a consistent upward flower angle.

### Experimental conditions

We used bumblebees (*Bombus terrestris*, L.) as model pollinators to examine the ability of bees to gather pollen in two entomophilous plants with different flower angles. Two captive colonies of naïve bees (one for experiments with snowdrop and another one for lesser celandine) were obtained from Koppert© (Nové Zámky, Slovakia) and were kept at 22–24°C in a room lit by natural light and neon light (370 lx). The bees were connected to a 90 × 50 × 40 cm insectarium by a plastic mesh tube and daily fed exclusively with honey solution (water 60% and honey 40%). We avoided pollen feeding to prevent bee contamination with pollen grains to ensure that all pollen grains on their bodies are the product of experimental conditions (see below). The bees were individually tested in the insectarium. Trials started by insertion, placing one freshly collected snowdrop/lesser celandine in flower glass test tubes on the front of the terrarium, 3 cm away from the back wall. When the bee was feeding inside the flower for 5 seconds, it was quickly removed and fixed in 70% ethanol for further examination of pollen loads (see below). About 10–15% of the bees flew away from the flower earlier. All of these bees were removed from further experiments due to possible contamination by pollen grains. Each flower was used only once. If the bee did not feed on the flower after 5 minutes, the trial was terminated, and the bee was tested again on one of the following days. The trials took place 1 week after the colony arrived, in March (experiments with snowdrops) and April (experiments with lesser celandine) 2022. A colony of bees was used for snowdrops, and one for experiments with the lesser celandine. All experiments were performed indoors under the same conditions as described to maintain the colony.

### Laboratory experiments

**Figure 1.**
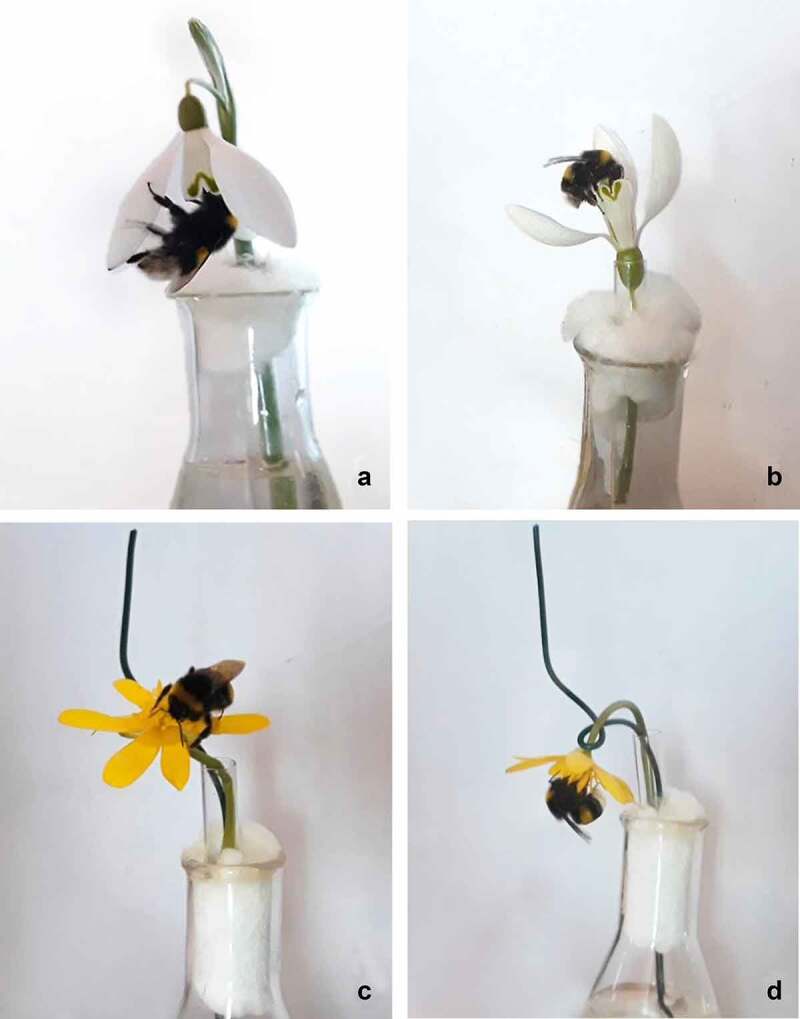
Examples of bumblebees visiting a) intact snowdrop flower with downward angle, b) treated snowdrop flower with upward angle, c) intact lesser celandine flower with upward angle and d) treated lesser celandine flower with upward angle in the laboratory.

To test the influence of flower position on the ability of insects to gather pollen, bees were randomly assigned to a group with unmanipulated flowers (downward for snowdrop [N = 21] and upward for lesser celandine [N = 22]) or to the treated group (upward for snowdrop [N = 19] and downward for lesser celandine [N = 21]) (Figure 1). The upward angle of the snowdrop was maintained by inserting a flower into a narrow glass tube (Figure 1b). Upward and downward angles in the lesser celandine was maintained by a wire (Figure 1c,d). The bees were stored in Eppendorf® microcentrifuge tubes with ethanol and removed within the next 10 days. Ethanol was filtered through filter paper and was placed in a laboratory dryer (Memmert UF30) for 10 min until it was dry. Pollen grains were removed from the surface of the filter paper by swabbing them with a cube (approximately 4 × 4 × mm square) of Fuchsin jelly^33^ using a clean entomological pin. The jelly was then placed on a slide glass and put in the laboratory dryer. After the jelly melted, the drop (liquid jelly) was covered with a coverslide. The pollen grains were immediately counted under the optical microscope^34–36^ LEICA DM 200LED (40× total magnification).

### Field experiments

We experimentally treated snowdrops (N = 25) and lesser celandine (N = 30) flowers to test the possible reproductive costs of flower angle in the field. Control flowers (N = 60 snowdrop and N = 30 lesser celandine) were not treated. The upward angle in the snowdrop was maintained by tying a flower with a twine on a stake. Lesser celandines were treated with a wire identically as in laboratory conditions. These two species coexist in the same localities but do not bloom simultaneously. We investigated snowdrops in a deciduous forest Jarovská bažantnica near Bratislava city (48°08ʹN, 17°.09ʹE). Lesser celandines were investigated in the edge of Trnava city (48°23ʹN, 17°34ʹE) due to practical reasons (this species has not so much restricted occurrence as snowdrop). Only one flower per plant was chosen to ensure non-independence of collected data. The plants were visited after 14 days (lesser celandine) or 7 weeks (snowdrop). For the lesser celandine, all seeds were removed, counted, and weighed in the laboratory with an electronic balance ABS 80–4 N on the nearest 0.0001 g. For the snowdrop, reduced sample sizes (below) did not allow us to make serious statistical comparisons, thus we compared capsule weight between upward and downward flowers. We acknowledge that this comparison is not definitive, and caution must be taken when interpreting these data.

### Statistical analyses

The number of pollen grains was defined as a dependent variable in a Generalized Linear Model (GLM) with Poisson data distribution and with identity function. Treatment (upward vs downward angle) and plant species were categorical predictors. Median values for capsule weights between upward and downward flowers in the snowdrop were compared with the Mann–Whitney U-test. Mean values for seed weights between upward and downward flowers in the lesser celandine were compared with an analysis of covariance, where the total number of seeds per plant was defined as a covariate. All statistical tests were performed with SPSS ver. 26.

## Results

### Pollen count

Snowdrops released more pollen grains on bees’ bodies than did lesser celandine flowers (GLM, F_1,79_ = 32.04, P < .001). Flowers with a downward angle released more pollen grains on bees’ bodies than flowers with an upward angle (GLM, F_1,79_ = 35.99, P < .001). The interaction term between the variables was significant (GLM, F_1,79_ = 36.58, P < .001), suggesting that downward snowdrops released significantly more pollen grains on bees’ bodies than upward snowdrops as well as the upward and downward lesser celandines’ flowers (contrast analyses, all P < .001) (Table 1). There were no differences between pollen grains released from upward snowdrops and upward and downward lesser celandine flowers on bees’ bodies (contrast analyses, all P > .4).

**Table 1.** Median values (± 95% CI) for pollen grains attached to bees’ bodies for plant species and flower angle.

	Snowdrop	Lesser celandine
Downward	404 (437.01–1408.32)	26 (22.43–78.81)
Upward	6 (6.54–44.2)	18.5 (-8.23–116.69)

### Reproductive success in the field

Wild animals (probably roe deer) removed 14 of 25 stakes with snowdrop flowers. Out of the remaining 11 snowdrops, five produced capsules (all contained diaspores), and the remaining six flowers (54%) aborted. This reduction of the sample does not allow us to make detailed statistical comparisons, but we consider some descriptive comparisons to be informative. Control flowers showed similar rates of abortions (35 out of 60, 58%) to experimental snowdrop flowers (Fisher's exact test, P = .53). There were no differences in the weight of capsules between experimentally treated upward snowdrop flowers (median = 0.65 g, 95% CI 0.28–1.24, N = 5) and untreated control (downward) flowers (median = 0.51 g, 95% CI 0.42–0.69, N = 25) (M-W U-test, U = 38, P = .18).

With respect to lesser celandine, after controlling for total number of seeds per each flower, untreated, upward flowers produced significantly heavier seeds (median = 0.017, 95% CI 0.014–0.023, N = 25) than experimentally treated, downward flowers (median = 0.014, 95% CI 0.012–0.017, N = 26) (ANCOVA, treatment: F_1,48_ = 4.62, P = .04, number of diaspores, F_1,48_ = 2.64, P = .11). None of the flowers were aborted, although the viability of apparently small seeds is not clear.

## Discussion

The adaptive significance of the downward-facing snowdrop flowers was unclear because they did not enhance pollinator attraction.^25^ Here, we show that bees gathered significantly more pollen grains from downward snowdrop flowers than upward flowers under controlled conditions, thus maximizing pollen dispersal to conspecific plants.

We recorded enhanced pollen loads on bee’s bodies in upward and downward flowers by counting pollen grains deposited on pollinator bodies, although previous studies examined pollen grains remaining in visited flowers.^9,10,17–19^ We consider these methods complementary, showing that downward flowers are more efficient in transferring pollen grains on pollinator bodies than upward flowers, but further research is necessary to determine whether stigmatic pollen loads^9,20^ in the snowdrop are influenced by flower angle.

A downward angle was the more efficient area for gathering pollen grains in the snowdrop than an upward angle. If gravity could increase the pollen export efficiency in downward flowers, it may also decrease the possibility of pollen deposited into stigmas. Snowdrops could counter these costs by producing a higher number of pollen loads. The same was not true, however, for the lesser celandine. Lesser celandine flowers show an upward angle, but pollen gathered by bees in this species was not affected by the experimentally induced downward angle. Pollen deposition strategies in upward lesser celandine flowers are probably mediated by pollen adhesion via pollenkitt or echinate surface structures or by electrical charge.^37–39^

Field experiments revealed that experimentally altered upward snowdrop flowers did not have a reduced weight of capsules, although these data must be interpreted with caution due to the reduced sample size. Lin and Forrest^14^ failed to detect any differences in the seed set of experimentally altered upward flowers of *Mertensia fusiformis*, suggesting that female fitness must not be influenced by flower angle. The spring of 2022 was exceptionally dryer compared to other years. Thus, although rainfall causes damage to reproductive organs,^10^ the absence of rain could enhance female fitness. In other words, it is possible that under high pollinator availability conditions, an upward flower angle could benefit both species in terms of more significant pollen receipt than flowers with downward angle. Differences in flower color would also contribute to pollinator visitation because, for instance, yellow flowers are more attractive to pollinators under certain circumstances than white flowers.^40^ Consider that roe deers damaged only stakes; thus, upward snowdrops were not targets of their feeding behavior and all untreated, downward flowers remained intact.

Some alternative hypotheses exist for the evolution of downward orientation for flowers blooming in early spring and for radially symmetric flowers. For example, in *Lonicera*, the early flowering species tend to have downward flowers, which is suggested to increase the floral temperature.^41^ Floral temperatures of snowdrops were unexpectedly cooler than the ambient air,^42^ which does not support this hypothesis. However, no study investigated differences in temperatures of upward and downward snowdrop flowers, which remains this alternative still open. In *Geranium refractum*, the downward orientation was found to exclude the visits by inferior pollinators but encourage the visits by effective pollinators.^43^ This explanation is also less probable in the case of snowdrops, where pollinators are scarce and adaptation to a diverse range of species is more expected than specialization.

The experimental inducement of downward flower angle in the lesser celandine was costly in terms of reduced seed mass. Downward flowers may also be less frequently visited by pollinators,^10,16^ and self-ed flowers can produce smaller seeds than outcrossed ones.^44,45^ These explanations are likely to explain the compromised reproductive success of the experimental downward lesser celandine flowers.

## Conclusion

Snowdrop with radially symmetrical, downward flowers solve trade-offs between pollinator attraction and effective pollen transfer on pollinators, thereby maximizing pollen dispersal to conspecific plants. This trade-off is reinforced by competition for pollinators that are scarce under unfavorable, cold conditions in early spring. High loads of pollen gathered by pollinators approaching downward flowers may simply be enhanced by gravity. The downward angle itself, however, does not enhance bees’ abilities to gather more pollen grains because experimentally treated downward flowers of the unfamiliar lesser celandine did not result in more effective pollen transfer than untreated, upward flowers. Further research should determine whether stigmatic pollen loads are affected by flower angle in snowdrops.

## References

[1] Rosas-Guerrero V, Aguilar R, Martén-Rodríguez S, Ashworth L, Lopezaraiza- Mikel M, Bastida JM, Quesada M, Irwin R. A quantitative review of pollination syndromes: do floral traits predict effective pollinators? Ecol Lett. 2014;17:388–6. doi:10.1111/ele.12224. 24393294

[2] Kelly D, Sork VL. Mast seeding in perennial plants: why, how, where? Annu Rev Ecol Syst. 2002;33:427–447. doi:10.1146/annurev.ecolsys.33.020602.095433.

[3] Fenster CB, Armbruster WS, Wilson P, Dudash MR, Thompson JD. Pollination syndromes and floral specialization. Annu Rev Ecol Evol Syst. 2004;35:375–403. doi:10.1146/annurev.

[4] Caruso CM, Eisen KE, Martin RA, Sletvold N. A meta‐analysis of the agents of selection on floral traits. Evolution. 2019;73(1):4–14. doi:10.1111/evo.13639. 30411337

[5] Dellinger AS. Pollination syndromes in the 21st century: where do we stand and where may we go? New Phytol. 2020;228(4):1193–1213. doi:10.1111/nph.16793. 33460152

[6] Roddy AB, Martínez‐Perez C, Teixido AL, Cornelissen TG, Olson ME, Oliveira RS, Silveira FA. Towards the flower economics spectrum. New Phytol. 2021;229(2):665–672. doi:10.1111/nph.16823. 32697862

[7] Ushimaru A, Kawase D, Imamura A. Flowers adaptively face down-slope in 10 forest-floor herbs. Funct Ecol. 2006;20:585–591. doi:10.1111/j.1365-2435.2006.01153.x.

[8] Aguilar‐García SA, Figueroa‐Castro DM, Valverde PL, Vite F, López‐Ortega G, Pérez‐Hernández MA. Reproductive biology of *Myrtillocactus geometrizans* (Cactaceae) flowers with contrasting orientation. Plant Spec Biol. 2022;37(3):243–256. doi:10.1111/1442-1984.12371.

[9] Yu YM, Li XX, Xie D, Wang H, Scopece G. Horizontal orientation of zygomorphic flowers: significance for rain protection and pollen transfer. Plant Biol. 2021;23:156–161. doi:10.1111/plb.13197. 33073503

[10] Nakata T, Rin I, Yaida YA, Ushimaru A, Arista M. Horizontal orientation facilitates pollen transfer and rain damage avoidance in actinomorphic flowers of Platycodon grandiflorus. Plant Biol. 2022;24(5):798–805. doi:10.1101/2021.09.08.459386. 35289975

[11] Huang SQ, Takahashi Y, Dafni A. Why does the flower stalk of *Pulsatilla cernua* (Ranunculaceae) bend during anthesis? Am J Bot. 2002;89:1599–1603. doi:10.3732/ajb.89.10.1599. 21665586

[12] Aizen MA. Down-facing flowers, hummingbirds and rain. Taxon. 2003;52:675–680. doi:10.2307/4135540.

[13] Chen JG, Yang Y, Zhang ZQ, Niu Y, Sun H. A nodding capitulum enhances the reproductive success of *Cremanthodium campanulatum* (Asteraceae) at high elevations in the Sino–Himalayan mountains. Plant Ecol Divers. 2013;6(3–4):487–494. doi:10.1080/17550874.2012.702793.

[14] Lin SY, Forrest JR. The function of floral orientation in bluebells: interactions with pollinators and rain in two species of *Mertensia* (Boraginaceae). J Plant Ecol. 2019;12(1):113–123. doi:10.1093/jpe/rtx073.

[15] Fulton M, Hodges SA. Floral isolation between *Aquilegia formosa* and *Aquilegia pubescens*. P Royal Soc Lond B Bio. 1999;266:2247–2252. doi:10.1098/rspb.1999.0915.

[16] Ushimaru A, Hyodo F. Why do bilaterally symmetrical flowers orient vertically? Flower orientation influences pollinator landing behaviour. Evol Ecol Res. 2005;7:151–160.

[17] Ushimaru A, Dohzono I, Takami Y, Hyodo F. Flower orientation enhances pollen transfer in bilaterally symmetrical flowers. Oecologia. 2009;160:667–674. doi:10.1007/s00442-009-1334-9. 19333624

[18] Wang Y, Meng LH, Yang YP, Duan YW. Change in floral orientation in *Anisodus luridus* (Solanaceae) protects pollen grains and facilitates development of fertilized ovules. Am J Bot. 2014;97(10):1618–1624. doi:10.3732/ajb.1000010. 21616797

[19] Castellanos MC, Wilson P, Thomson JD. ‘Anti-bee’ and ‘pro-bird’ changes during the evolution of hummingbird pollination in *Penstemon* flowers. J Evolution Biol. 2004;17:876–885. doi:10.1111/j.1420-9101.2004.00729.x. 15271088

[20] Nevard L, Vallejo‐Marín M. Floral orientation affects outcross pollen deposition in buzz‐pollinated flowers with bilateral symmetry. Am J Bot. 2022:11. doi:10.1002/ajb2.16078. PMC9828177 36193950

[21] Naghiloo S, Bellstedt DU, Claßen-Bockhoff R. Pollination biology in *Roepera*(Zygophyllaceae): how flower structure and shape influence foraging activity. PlantSpec Biol. 2020;35:72–80. doi:10.1111/1442-1984.12262.

[22] Stewart AB, Diller C, Dudash MR, Fenster CB. Pollination-precision hypothesis: support from native honey bees and nectar bats. New Phytol. 2022;235:1629–1640. doi:10.1111/nph.18050. 35194792

[23] Culbert BM, Forrest J. Floral symmetry affects bumblebee approach consistency in artificial flowers. J Poll Ecol. 2016;18:1–6. doi:10.26786/1920-7603(2016)10.

[24] Armbruster WS, Muchhala N. Floral reorientation: the restoration of pollination accuracy after accidents. New Phytol. 2020;227:232–243. doi:10.1111/nph.16482. 32252125

[25] Wang H, Tie S, Yu D, Guo Y-H, Yang C-F, Gronenberg W. Change of floral orientation within an Inflorescence affects pollinator behavior and pollination efficiency in a Bee-Pollinated plant, *Corydalis sheareri*. PLoS ONE. 2014;9(4):e95381. doi:10.1371/journal.pone.0095381. 24743567 PMC3990675

[26] Prokop P, Zvaríková M, Ježová Z, Fedor P. Functional significance of flower orientation and green marks on tepals in the snowdrop *Galanthus nivalis* (Linnaeus, 1753). Plant Sig Beh. 2020;15(11):1807153. doi:10.1080/15592324.2020.1807153. PMC7588181 32799622

[27] Cox F. Gardener’s guide to Snowdrops. Ramsbury (UK): The Crowood Press Ltd; 2013. 256.

[28] Chudzik B, Snieżko R, Szaub J. Biology of flowering of *Galanthus nivalis* L. (Amaryllidaceae). Ann Univ Mariae Curie-Sklodowska EEE Hortic. 2002:1–10. https://agris.fao.org/agris-search/search.do?recordID=PL2007000421.

[29] Taylor K, Markham B. *Ranunculus Ficaria* L. (Ficaria verna Huds.; F. Ranunculoides Moench). J Ecol. 1978;66(3):1011–1031. doi:10.2307/2259310.

[30] Sell PD. Ranunculus ficaria L. sensu lato Watsonia. 1994;20:41–45.

[31] Marsden-Jones EM. Ranunculus ficaria Linn.: life-history and pollination. J Linn Soc Lond Bot. 1935;50:39–55. doi:10.1111/j.1095-8339.1935.tb01501.x.

[32] Marsden-Jones EM, Turrill WB. Studies on ranunculus ficaria. J Genet. 1952;50:522–534. doi:10.1007/BF02986847.

[33] Kearns CA, Inouye DW. Techniques for pollination biologists. Niwot, Colorado, USA: University Press of Colorado; 1993. p. 583.

[34] Forup ML, Memmott J. The restoration of plant–pollinator interactions in hay meadows. Restor Ecol. 2005;13(2):265–274. doi:10.1111/j.1526-100X.2005.00034.x.

[35] Alarcón R. Congruence between visitation and pollen-transport networks in a California plant-pollinator community. Oikos. 2010;119:35–44. doi:10.1111/j.1600-0706.2009.17694.x.

[36] Howlett BG, Walker MK, Rader R, Butler RC, Newstrom-Lloyd LE, Teulon DAJ. Can insect body pollen counts be used to estimate pollen deposition on pak choi stigmas. N Z Plant Protect. 2011;64:25–31. doi:10.30843/nzpp.2011.64.5951.

[37] Hesse M, Ulrich S. Pollen: stunning diversity and amazing beauty. Biol Zeit. 2012;42:34–41.

[38] Lin H, Gomez I, Meredith JC. Pollenkitt wetting mechanism enables species-specific tunable pollen adhesion. Langmuir. 2013;29:3012–3023. doi:10.1021/la305144z. 23402563

[39] Vaknin Y, Gan-Mor S, Bechar A, Ronen B, Eisikowitch D. The role of electrostatic forces in pollination. Plant Syst Evol. 2000;222:133–142. doi:10.1007/BF00984099.

[40] Kay QON. Preferential pollination of yellow-flowered morphs of *Raphanus raphanistrum* by *Pieris* and *Eristalis* spp. Nature. 1976;261(5557):230–232. doi:10.1038/261230a0.

[41] Xiang GJ, Guo YH, Yang CF. Diversification of floral orientation in *Lonicera* is associated with pollinator shift and flowering phenology. J Syst Evol. 2021;59(3):557–566. doi:10.1111/jse.12554.

[42] Rejšková A, Brom J, Pokorný J, Korečko J. Temperature distribution in light-coloured flowers and inflorescences of early spring temperate species measured by infrared camera. Flora. 2010;205:282–289. doi:10.1016/j.flora.2009.05.001.

[43] Wang H, Xiao CL, Gituru RW, Xiong Z, Yu D, Guo YH, Yang CF. Change of floral orientation affects pollinator diversity and their relative importance in an alpine plant with generalized pollination system, Geranium refractum (Geraniaceae). Plant Ecology. 2014;215(10):1211–1219. doi:10.1007/s11258-014-0379-y.

[44] Galen C, Plowright RC, Thomson JD. Floral biology and regulation of seed set and seed size in the lily, *Clintonia Borealis*. Am J Bot. 1985;72(10):1544–1552. doi:10.1002/j.1537-2197.1985.tb08418.x.

[45] Lu J, Yi H, Tan D, Baskin CC, Baskin JM. Germination of seeds from flowers along a continuum of long to short styles in the cold desert perennial herb *Ixiolirion songaricum*. Plants. 2022;11(11):1452. doi:10.3390/plants11111452. 35684225 PMC9182588

